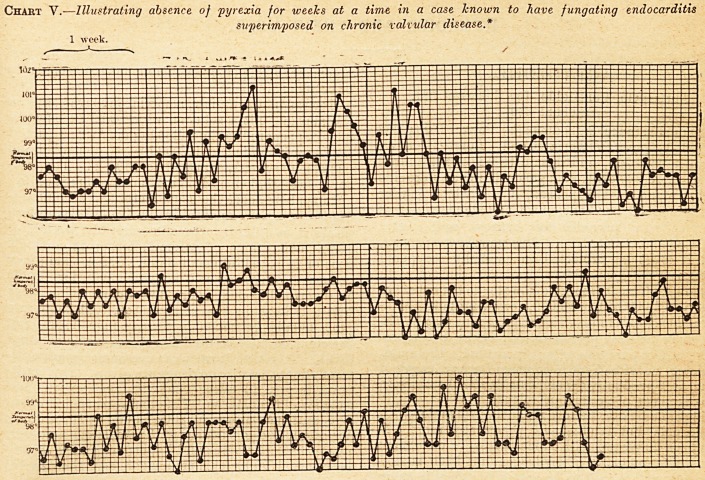# Fungating Endocarditis and the Possibility of Its Presence without Pyrexia

**Published:** 1907-02-23

**Authors:** 


### SPECIAL ARTICLE.

### FUNGATING ENDOCARDITIS AND THE POSSIBILITY OF ITS PRESENCE WITHOUT PYREXIA.

There is still a widespread belief that all fungating endocarditis cases present well-marked and unmistakable clinical symptoms, and that they may be put into one or other of two main groups, namely: (*a*) Those which resemble ordinary pyæmia—the *septic* or *pyæmic* type; and (*b*) those which resemble a prolonged specific fever such an enterica—the *typhoid* type. This has long been the teaching of text-books; but there is no doubt that both the pyæmic and the typhoid types are much rarer than they used to be; that the majority of cases seen nowadays are not obviously pyæmic; that a minority may resemble typhoid fever; and that the bulk of the cases present most of the features of cardiac disease with failure of compensation, the chief difficulty in diagnosis being to decide whether the failure is purely mechanical or whether there is additional trouble in the form of fungating endocarditis. In short, most of the cases are distinctly *cardiac* in type. The reason why the pyæmic forms are rarer than they used to be is no doubt dependent upon the comparative rarity of pyæmia itself. Antisepsis and asepsis in surgery and gynæcology are to be thanked for this. In former days, when surgical pyæmia was common, numbers of heart-valves showed fungating endocarditis at the autopsy; clinically these cases were for the most part pyæmia pure and simple, the septic endocarditis being a secondary accident which often gave rise to no particular symptoms other than those due to the pyæmia itself. The disappearance of most of these cases is paralleled by the comparative rarity of lardaceous disease nowadays. Formerly, when prolonged suppuration was the rule rather than the exception in surgery, there would be as many as one or two cases of lardaceous disease in the post-mortem room of a large hospital every week; now there may not be an autopsy upon a case of lardaceous disease once in three months in the same hospital, and then it is more likely to be in a case of phthisis than in one of surgical suppuration.

It is true that some cases of fungating endocarditis may be quite anomalous, there being little or nothing to point to cardiac trouble at all. For example, a male patient, aged about 25, recently presented himself, complaining of vomiting blood. At first the condition seemed to be one of gastric ulcer, but on inquiry it was found that he had had recurrent and severe epistaxis on several occasions during the previous three weeks, and on further examination blood was found trickling from both ears, which had long been affected by chronic otitis media. The vomited blood was no doubt partly swallowed, but so much was brought up that part at least must have been gastric. No other site of bleeding was found. There was no pyrexia; an

Chart I.—*Illustrating Prolonged Pyrexia in Fungating Endocarditis.*

indefinite cardiac bruit was heard; but the spleen was palpable, and the blood-count showed there was no primary blood disease. Upon the strength of three sites of spontaneous hæmorrhage—ears, nose, and stomach—a source for sepsis in the otitis media, a palpable spleen and a cardiac bruit, a diagnosis of fungating endocarditis was hazarded, and ultimately confirmed by autopsy.

Notwithstanding the occurrence of anomalous cases of this sort, we must repeat that the majority of cases present in the main distinctly cardiac symptoms. Very often there is a clear history of acute rheumatism, chorea, or syphilis to account for an organic valvular lesion; the patient comes up complaining of shortness of breath, pallor, weakness, or œdema; and, supposing the case to be, for example, aortic regurgitation, it is often extremely difficult to say whether the symptoms are due entirely to the mechanical effects of this valve lesion, or whether there is fungating endocarditis as well. Upon what points should one lay stress in deciding the question?

The presence of pyrexia will always arouse suspicion of fungating endocarditis in such a case. If in a case of chronic valvular heart disease there is persistent pyrexia without any obvious cause for it, such as acute pleurisy, tonsillitis, or actual rheumatic fever, there must always be strong suspicion that fungating endocarditis has been superposed upon the fibrotic affection of the valves; sometimes the condition is by no means very virulent, and the end may be long delayed. The following is an example of a case which lasted over four months:—There had been 31 days' definite illness before the record of her temperature was known, and then for 89 days there was almost continuous pyrexia, as shown in Chart I., page 372.

The patient died; during life the bruits suggested old-standing lesions of both aortic and mitral valves; these were confirmed by autopsy, and upon the top of the chronic valve lesions there were, as was expected, large masses of recent fibrin.

It is, of course, possible for fungating endocarditis to get well; the prognosis is extremely grave, but the possibility of recovery is proved by the occurrence at autopsies from time to time of healed perforations in valves, organised and calcareous masses attached to the free edges of valves, and minute aneurysms on valves. The following is the temperature chart (Chart II.) of a case in which there were bruits indicative of a mitral lesion of rheumatic origin, and in which there were other evidences of fungating endocarditis such as multiple emboli, and enlargement of the spleen, which will be discussed below. Clinically there was no doubt that this patient had fungating endocarditis; life was despaired of for weeks, the illness was so severe, and the cardiac trouble so great; but ultimately complete recovery, with compensation of the mitral lesion, resulted:—

CHART II.—*Illustrating the long duration of a case of fungating endocarditis of the cardiac type; with persistent pyrexia.*

It is important to know, however, that *the absence of pyrexia does not exclude the possibility of fungating endocarditis.* Presumably the virulence of the organism present varies greatly in different cases, and a less virulent organism is less likely, one supposes, to cause pyrexia than is one which is more virulent. The number of organisms present is also very variable, and presumably there would be less pyrexia if there were fewer organisms. In other words, there are very different degrees in the severity of the affection in different cases. Over and above this, however, there is a factor upon which too little stress is laid in text-books, and this is that *persons suffering from valvular heart disease of long standing are very apt to have persistently subnormal temperatures.* The following is a typical temperature chart of such a case, in which the usual temperature was about 97° F. instead of 98.4° F.: —

Chart III.—*Illustrating the persistently subnormal temperature in a case of chronic mitral disease.*

This reduction in the average temperature of many persons suffering from chronic valvular disease is very important; for in the case just mentioned a temperature of 98.4° F. might have attracted no attention, and yet would have been slight pyrexia for this particular patient. A temperature of 99° F. in him would correspond to a temperature of 100.4° F. in a healthy person, and so on. Quite slight degrees of pyrexia in cases of chronic valvular heart disease may therefore be of much greater significance than they are in other people. Chart IV. is an illustration of this: the patient was suffering from aortic disease of long standing, with failure of compensation. At the autopsy there were fungating masses of fibrin on the aortic valves, though the temperature during life had been mostly subnormal. When opportunity arises for recording the temperature in such cases for weeks and months before death it will usually be found that now and then there is definite, even if only slight, pyrexia; but that for many consecutive weeks there may be none at all, is shown by chart V., page 375, from a case of chronic mitral disease, in which the occurrence of emboli, etc., had led to the diagnosis of fungating endocarditis seventeen weeks before it was confirmed by autopsy.

Chart IV.—*Illustrating the slight degree of pyrexia there may be in a case of chronic valvular disease with terminal fungating endocarditis.*

We have entered into the question of pyrexia as a sign of fungating endocarditis at some length, because, although the presence of pyrexia is a very valuable aid in making a positive diagnosis, we wish to make it clear that the absence of pyrexia does not negative this conclusion. It remains to discuss briefly the other symptoms and signs which make one suspect that a patient with chronic valvular disease of the heart has a superimposed fungating endocarditis.

*Enlargement of the spleen* is a very suspicious sign. In chronic simple heart disease the spleen, contrary to what might be expected, seldom becomes large enough to be palpable an inch or more below the ribs. In fungating endocarditis, on the other hand, the spleen is almost always palpably enlarged, even if there be no evidence of infarction; whilst with infarction the enlargement may be quite considerable.

*Progressive anæmia* is another important sign. This is particularly useful in the case of mitral stenosis, for here, if the heart lesion be "simple," the face is far from anæmic, but is flushed, particularly over the malar bones. Should such a case become progressively anæmic without other obvious cause, such as menorrhagia or the like, fungating endocarditis is more than probable. In aortic cases the sign is of less certain value, because a typical "simple" aortic case is usually anæmic, or at least pale, quite apart from fungating endocarditis.

*Multiple emboli* in a heart case are a not very uncommon, and almost pathognomic, sign of fungating endocarditis. A single embolus has not the same significance, for it is well known that cerebral embolism, for example, may occur in otherwise uncomplicated, and apparently compensated, cases of mitral stenosis. If, however, a sudden acute pain over the splenic area should be followed in a day or two by another sudden pain in one loin, and hæmaturia, the evidence of the second embolus succeeding the first so closely would almost certainly prove that the chronic heart case had developed fungations on the valves. Embolism is, of course, by no means restricted to the organs in which infarcts occur; it may take place in the liver, the stomach, the bowel, an upper or lower limb, in a branch of the facial artery, in fact anywhere except the lungs. An infarct in the lungs is seldom evidence of fungating endocarditis, because the fungating masses are so much rarer on the valves of the right side of the heart than they are upon those of the left.

*A sudden complete change in the character of the bruit heard* is occasionally the first sign that may lead to a suspicion of fungating endocarditis. For example, a man came complaining that his wife could not sleep at night, being kept awake by a musical sound he had in his chest. This was found to be a tremendously loud musical aortic bruit, diastolic in time. The man himself seemed quite well. A few days later the bruit suddenly lost its musical character, and was now barely audible with the

CHART V.—*Illustrating absence of pyrexia for weeks at a time in a case known to have fungating endocarditis superimposed on chronic valvular disease.**

stethoscope. So sudden and radical a change in the bruit suggested a sudden and radical change in the condition of the valve; fungating endocarditis seemed to be the only cause which could bring this about so suddenly and apparently spontaneously; the diagnosis was made accordingly, and its correctness was verified at autopsy in due course.

*Spontaneous hæmorrhages* in a heart case are very suggestive. An instance of this has already been given. It is rare for a simple valvular heart disease to cause hœmorrhages *other than hæmoptysis or menorrhagia.* Should petechiæ appear under the skin, or blood in the urine, a suspicion of fungating endocarditis at once arises.

*Ophthalmoscopic examination* is not infrequently helpful. In simple valvular disease it is almost unknown to get optic neuritis or retinal hæmorrhage. If, therefore, it is certain that the patient's condition is due primarily to valvular disease of the heart, and not, *e.g.*, to granular kidney, the presence of optic neuritis or of retinal hæmorrhages will indicate that fungating endocarditis has supervened.

The question may be asked, Would not a blood-count assist the diagnosis? It might be expected that a condition in which organisms were growing in the masses of fibrin on the heart valves would lead to a leucocytosis; this, however, is not as a rule the case. Generally the leucocytes do not exceed 15,000 per cub. mm., so that a leucocyte count affords no reliable evidence either for or against fungating endocarditis. There is one way, however, in which a blood examination might be of assistance, and that is to collect a few c.c. of blood from a vein aseptically, and have it cultivated for micro-organisms. Should other microbes than staphylococci be present (staphylococci are usually an accidental contamination from the skin) the diagnosis will be almost certain, though negative evidence from cultivation would not prove the converse.

To sum up, therefore, we would say that though there are cases of fungating endocarditis which conform to the old classification into the pyæmic and the typhoid types, and though there are some which are quite anomalous, the majority of the cases seen nowadays are of the cardiac type, cases, that is to say, in which the heart disease is of long standing, and in which, when symptoms of failure set in, it is difficult to decide whether these are purely mechanical or whether there may not be due to a superimposed fungating endocarditis. The presence of pyrexia without obvious cause is a strong indication of the latter; but some cases of fungating endocarditis may have little or no pyrexia; perhaps this in part depends upon the depression of the normal temperature in chronic heart cases. The main points to be on the look out for as aids in the diagnosis are enlargement of the spleen, progressive anæmia, multiple emboli, sudden and complete changes in the character of the bruit or bruits, spontaneous hæmorrhages other than hæmoptysis and menorrhagia, the presence of optic neuritis or retinal hæmorrhages, and finally the presence of micro-organisms in cultivations made from the patient's blood.

* It should be mentioned that in all these cases the temperatures were recorded four-hourly; but in the charts reproduced here only the highest and the lowest temperatures for each twenty-four hours are given.

## Figures and Tables

**Chart I. f1:**
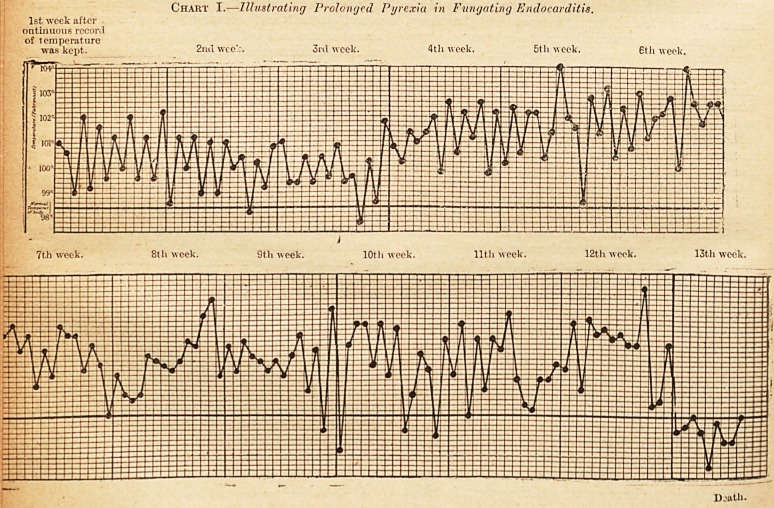


**Chart II. f2:**
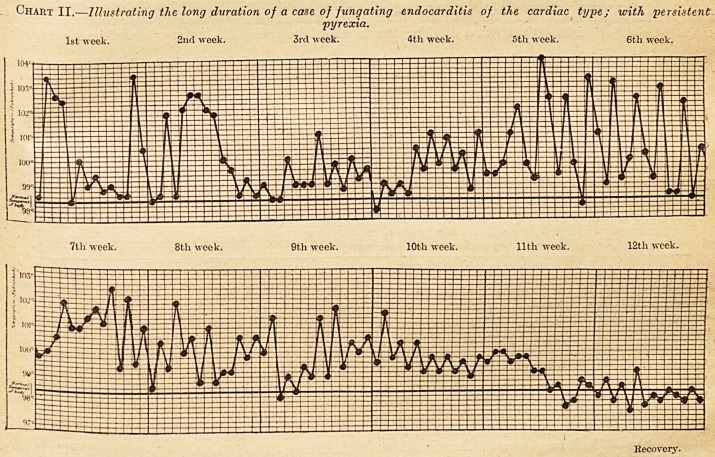


**Chart III. f3:**
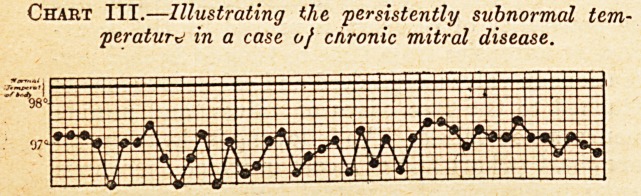


**Chart IV. f4:**
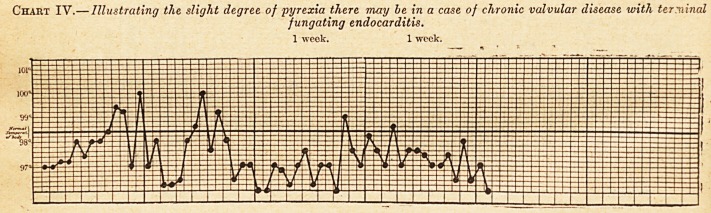


**Chart V. f5:**